# Supplementary material for: Three-dimensional stochastic simulation of chemoattractant-mediated excitability in cells
Source: PLoS Comput Biol. 2021 Jul 14;17(7):e1008803. doi: 10.1371/journal.pcbi.1008803 (PMC8330952; doi:10.1371/journal.pcbi.1008803)
Supplement: S1 File — (PDF) [file pcbi.1008803.s010.pdf]

Three-dimensional stochastic simulation of chemoattractant-mediated excitability in cells

Supplementary Information

Debojyoti Biswas[1], Peter N. Devreotes[2], Pablo A. Iglesias[1,2,3]

[1]Department of Electrical & Computer Engineering, Johns Hopkins University Whiting School of Engineering, Baltimore, Maryland, USA
[2]Department of Cell Biology, Johns Hopkins University School of Medicine Baltimore, Maryland, USA
[3]Author to whom correspondence should be addressed (pi@jhu.edu)

1 Specifying reactions in URDME

Here we demonstrate the process of specifying reactions in particular to our system. Additional details can be found in the Github page for URDME (https://github.com/URDME/urdme/blob/master/doc/manual.pdf). We used a 3D spherical shell to mimic the cell shape where we defined the nodes on the outer surface as part of the "membrane" subdomain whereas the interior as the "cortex" subdomain. To restrict the diffusion of the molecules of exclusively membrane bound species, for example, receptors to the cortex, the entries in the diffusion matrix correspond to the cortex elements were manually made zero.

As an example of how to translate the reactions of Tables 1–4 into the URDME code, we consider reaction 1 from Table 1. It is given by,

$$\text{H} + \text{cAMP} \rightarrow \text{H:C},$$

with propensity function

$$k_H[\text{cAMP}][\text{H}].$$

In this case, the reaction is written:

```
gpcr1  = 'H + cAMP  > kH*H*cAMP/vol   > HC';
```

where kH = $\frac{k_H}{k}$ and $k = N_{\text{Avogadro}} \times 10^{-21}$ is a conversion factor from μM units into #molec/μm^3. Note that gpcr1 is the label for this reaction. Because k_H is in units of $\mu\text{M}^{-1}\text{s}^{-1}$, the extra "vol" term is introduced. This represents the volume of the macroelements (nodes).

An example of an enzymatic reaction is no. 2 in Table 4. The reaction is

$$\text{RasGTP} \xrightarrow{\text{PKB}^*\text{s}} \text{RasGDP},$$

with propensity function

$$a_2[\text{RasGTP}][\text{PKB}^*\text{s}].$$

In this case, the reaction is written as:

```
en2  = 'RasGTP > a2*RasGDP*PKBsa/vol > RasGDP';
```

In this case, PKB*s (PKBsa) does not appear in the left side as in the previous example because it acts as an enzyme in the conversion reaction from RasGTP to RasGDP.

When considering the effect of a spatially-differentiated thresholds, we used different values for the reaction constant a_2 (a_{20}, a_{21}) in en2 for different subdomains. In this context, we divided our simulation domain further into subdomains "basal" (sd = 0) and "apical" (sd = 1). In this case, the reactions were entered as follows.

In the basal domain:

```
en20  = 'RasGTP > sd == 0 ? a20*RasGDP*PKBsa/vol  : 0.0   > RasGDP'
```

and in the apical:

```
en21 = 'RasGTP > sd == 1 ? a21*RasGDP*PKBsa/vol : 0.0  > RasGDP'
```

Finally, an example of a generation reaction is no. 5 in Table 4. The reaction is

$$\varnothing \rightarrow \mathrm{PIP}_2,$$

with propensity function b_1. In this case, the reaction is written as:

```
en5 = '@ > b1*vol > PIP2';
```

The reaction is similar to a birth process where the symbol “@” implies the production is de novo and b_1 is in $\mu\mathrm{Ms}^{-1}$; the extra “vol” term is multiplied.

2 Implicit and explicit LEGI schemes

Though our study relies exclusively on stochastic descriptions of the reactions (as illustrated in the previous section), to explain the difference(s) between the “implicit” and “explicit” formalisms of the LEGI scheme we will use deterministic description based on PDEs.

The explicit dynamics (Fig. S3E,F) in the deterministic setup can be represented as follows. First, for the difference scheme:

$$\frac{\partial[X]}{\partial t} = k_1[I^*] - k_2[RR][X] + D_X\nabla^2[X] \tag{1a}$$

$$\frac{\partial[RR]}{\partial t} = k_0 + k_1[G_{\beta\gamma}] - k_2[RR][X] - k_3[RR] + D_{RR}\nabla^2[RR] \tag{1b}$$

Assuming a quasi-steady-state and a spatially-homogeneous solution, we obtain:

$$0 = k_1[I^*] - k_2[RR]_{ss}[X]_{ss} \tag{2a}$$

$$0 = k_0 + k_1[G_{\beta\gamma}] - k_2[RR]_{ss}[X]_{ss} - k_3[RR]_{ss} \tag{2b}$$

Replacing from 2a into 2b yields:

$$k_0 + k_1[G_{\beta\gamma}] = k_1[I^*] + k_3[RR]_{ss}$$

and solving for $[RR]_{ss}$ we obtain:

$$[RR]_{ss} = \frac{k_0}{k_3} + \frac{k_1}{k_3}([G_{\beta\gamma}] - [I^*])$$

Thus, we directly used the steady-state expression as the coupling term between LEGI and STEN as follows:

```
en3 = 'RasGDP > (a30+a31*(BG-I))/(a4*a4/(vol*vol)*PIP2*(PIP2-1)+1))*RasGDP  > RasGTP';
```

where BG = $G_{\beta\gamma}$ and Ia = I^*.

Similarly for the ratio scheme:

$$\frac{\partial[RR]}{\partial t} = k_0 + k_1[G_{\beta\gamma}] - k_2[RR][X] - k_3[RR] + D_{RR}\nabla^2[RR]$$

we obtain, at steady-state:

$$[RR]_{ss} = \frac{k_{10} + k_1[G_{\beta\gamma}]}{k_{20} + k_3[I^*]}. \tag{3}$$

The corresponding reaction in URDME is given by,

```
en3 = 'RasGDP >
(a3*(a30+BG)/(a30+Ia))/(a4*a4/(vol*vol)*PIP2*(PIP2-1)+1))*RasGDP  > RasGTP';
```

where BG = $G_{\beta\gamma}$ and Ia = I^*.

3 Kymograph generation

To create the cell perimeter kymographs, the nodes lying between the z sections between heights 1 and 2 μm (Fig. 4B), were projected on a plane, resulting in an annular section. This was then divided into 90 sectors each with a 4° span. The maximum value for the specific species over all the voxels in each sector was used as the representative one. Thus, at each time point we generated an array of 90 representative values. We continued this process over the intended period of time to generate the respective kymographs (Fig. 4D, S6 Fig.D, Fig. 6A-C, Fig. 7A,B, S7 Fig.F).

4 Parameter selection and parameter variation

Whenever the direct experimentally measured rate constants [1, 2] were available, we used the values in our model (Table 1). In other cases, we relied on some indirect approaches. For example, the experimental half times of $G_{\alpha 2\beta\gamma}$ association and dissociation were available [3], so we chose reaction rates, k_{E0}, k_E, k_{-E} (Table 2) to achieve the similar time constants from simulated response. The total number of G-protein ($G_{\alpha 2\beta\gamma}$+$G_{\beta\gamma}$) and the LEGI-inhibitor (I+I*) were assumed to have the 210,000 molecules on average.

The rest of the parameters of LEGI schemes were chosen to achieve similar mean level of steady-state values and adaptation time. The adaptation time was computed as the time taken by the mean nodal profile of RR to reach within 2% of the final steady-state value. The peak amplitude was computed as the maximum value of the smoothened mean nodal profile of RR and the peak time was the time taken to reach that after the stimulus was applied. Unlike the LEGI implicit difference and ratio scheme, in LEGI-AIF, the species X, Y and RR were explicitly modeled (reactions no. 7–15 in Table 3). When choosing the reaction rate constants for the LEGI-AIF scheme, we tried to suppress the oscillations in the mean profile of RR. For saturating dose of cAMP, the maximum value of the total number of intermediate X and Y were approximately 30,000 and 45,000, respectively.

For the selection of the parameter set for the excitable system, we relied on the experimentally-determined wave speed, qualitative and quantitative behaviors of different entities [3–7]. Most of the reactions in STEN module are single-step reactions except for the autocatalytic reaction (reaction 3 in Table 4). The complex propensity function of reaction 3, involves several steps: inhibition by PIP2, basal activation as well as RR-dependent activation. To reduce the computation burden of simulating these first/ second-order reactions as well as estimation of more number of unknown reaction parameters, we simplified this multistep reaction process using quasi-steady-state

assumption. PKBA, a component of PKB*s shuttle between membrane and cytosol, we assumed that inactivated PKBs is in the cytosol which on activation comes to the membrane as PKB*s. We did not model the activation/inactivation of PKBs explicitly, rather we assumed it as a birth/death process (reactions no. 9&10 in Table 4).

In the present study, we investigated the effect of the intrinsic noise of the system as well as the effect of the cell-to-cell variations. In the latter, we varied all the parameters (both reaction rates and the diffusion constants) following a Gaussian distribution with nominal values and standard deviations. All the nominal values and the standard deviations (typically 10%) are listed in the respective tables. We assumed homogeneity in the cell but heterogeneity across the cells in terms of the parameters.

5 Computational and Statistical analysis

All simulations were run using URDME package and custom codes written in MATLAB 2019a (MathWorks, Natick, MA, USA) on an macOS (version 10 and 11). The statistical analysis were performed with custom codes written in MATLAB 2019a (MathWorks, Natick, MA, USA) and RStudio 3.5.1 (RStudio, Boston, MA, USA). The statistical tests used, as indicated in this manuscript, were as follows: Mann-Whitney-Wilcoxon (MWW) test, Kolmogorov-Smirnov (K-S) test. For all tests, the significance level was set to less than 0.05.

References

[1] Van Haastert PJ, De Wit RJ. Demonstration of receptor heterogeneity and affinity modulation by nonequilibrium binding experiments. The cell surface cAMP receptor of *Dictyostelium discoideum*. J Biol Chem. 1984;259(21):13321–8.

[2] Ueda M, Sako Y, Tanaka T, Devreotes P, Yanagida T. Single-molecule analysis of chemotactic signaling in Dictyostelium cells. Science. 2001;294(5543):864–7. doi:10.1126/science.1063951.

[3] Tang M, Wang M, Shi C, Iglesias PA, Devreotes PN, Huang CH. Evolutionarily conserved coupling of adaptive and excitable networks mediates eukaryotic chemotaxis. Nat Commun. 2014;5:5175. doi:10.1038/ncomms6175.

[4] Xu X, Meier-Schellersheim M, Yan J, Jin T. Locally controlled inhibitory mechanisms are involved in eukaryotic GPCR-mediated chemosensing. J Cell Biol. 2007;178(1):141–53. doi:10.1083/jcb.200611096.

[5] Takeda K, Shao D, Adler M, Charest PG, Loomis WF, Levine H, et al. Incoherent feedforward control governs adaptation of activated Ras in a eukaryotic chemotaxis pathway. Sci Signal. 2012;5(205):ra2. doi:10.1126/scisignal.2002413.

[6] Huang CH, Tang M, Shi C, Iglesias PA, Devreotes PN. An excitable signal integrator couples to an idling cytoskeletal oscillator to drive cell migration. Nat Cell Biol. 2013;15(11):1307–1316. doi:10.1038/ncb2859.

[7] Miao Y, Bhattacharya S, Edwards M, Cai H, Inoue T, Iglesias PA, et al. Altering the threshold of an excitable signal transduction network changes cell migratory modes. Nat Cell Biol. 2017;19(4):329–340.
