# Supplementary material for: Three-dimensional stochastic simulation of chemoattractant-mediated excitability in cells
Source: PLoS Comput Biol. 2021 Jul 14;17(7):e1008803. doi: 10.1371/journal.pcbi.1008803 (PMC8330952; doi:10.1371/journal.pcbi.1008803)
Supplement: S2 File — Matlab scripts used for simulating the various models. (ZIP) [file pcbi.1008803.s011.zip › Code/urdme-master/doc/manual.pdf]

The URDME manual
Version 1.3

Pavol Bauer[1] Stefan Engblom[1]

March 14, 2017

[1] *Division of Scientific Computing, Department of Information Technology, Uppsala University, P. O. Box 337, SE-75105 Uppsala, Sweden, e-mail: stefane@it.uu.se.*

Manager of this release: Stefan Engblom (to whom correspondence should be addressed).

1 Introduction

Stochastic simulation methods are frequently used to study the behavior of cellular control systems modeled as continuous-time discrete-space Markov processes (CTMC). Compared to the most frequently used deterministic model, the reaction rate equations, the mesoscopic stochastic description can capture effects from intrinsic noise on the behavior of the networks [1, 9, 27, 28, 31].

In the discrete mesoscopic model the state of the system is the copy number of the different chemical species and the reactions are usually assumed to take place in a well-stirred reaction volume. The chemical master equation is the governing equation for the probability density, and for small to medium sized systems it can be solved by direct, deterministic methods [12, 13, 17, 23, 25]. For larger models however, exact or approximate kinetic Monte Carlo methods [19, 20] are frequently used to generate realizations of the stochastic process. Many different hybrid and multiscale methods have also emerged that deal with the typical stiffness of biochemical reactions networks in different ways, see [4, 7, 21, 24, 29] for examples.

Many processes inside the living cell can not be expected to be explained in a well-stirred context. The natural macroscopic model is the reaction-diffusion equation which has the same limitations as the reaction rate equations. By discretizing space with small subvolumes it is possible to model the reaction-diffusion process by a CTMC within the same formalism as for the well-stirred case. A diffusion event is now modeled as a first order reaction from a subvolume to an adjacent one and the state of the system is the number of molecules of each species in each subvolume. The corresponding master equation is called the reaction-diffusion master equation (RDME) and due to the very high dimensionality it cannot be solved by deterministic methods for realistic problem sizes.

The RDME has been used to study biochemical systems in [8, 16]. Here the next subvolume method (NSM) [8], an extension of Gibson and Bruck's next reaction method (NRM) [18], was suggested as an efficient method for realizing sample trajectories. An implementation on a structured Cartesian grid is freely available in the software MesoRD [22].

The method was extended to unstructured meshes in [14] by making connections to the finite element method (FEM). This has several advantages, the most notable one being the ability to handle complicated geometries in a flexible way. This is particularly important in cell-biological models where internal structures often must be taken into account.

This manual describes the software URDME which provides an efficient, modular implementation capable of stochastic simulations on unstructured meshes. URDME is easy to use for simulating and studying a particular model in an applied context, but also for developing and testing new approximate methods. We achieve this by relying on third-party software for the geometry definition, meshing, preprocessing and visualization, while using

a highly efficient computational core written in ANSI C for the actual stochastic simulation. This keeps the implementation of the Monte Carlo part small and easily expandable, while the user benefits from the advanced pre- and post-processing capabilities of modern FEM software. In this version of URDME, we provide an interface to Comsol Multiphysics 5.2a [5], but also to the earlier software PDE Toolbox [26], often available with standard Matlab university licenses.

The rest of this manual is organized as follows. Section 2 summarizes the major changes of the 1.3 release as well as the downloading and installation procedures. An overview of the software structure is presented in Section 3 and the details concerning the input to the code, the provided interface to Comsol and the way models should be specified are found in Section 4. An URDME model is set up and simulated in a step-by-step manner in Section 5 and in Section 6 we show how a new solver can be integrated into the URDME infrastructure.

In two appendices we recapitulate the mesoscopic reaction-diffusion model and show how the stochastic diffusion intensities are obtained from a FEM discretization of the diffusion equation. We also list for reference a few stochastic simulation algorithms.

We refer the interested reader to the paper [6] for further information on the URDME software, including comparisons to other available software and examples of some more advanced usage.

2 The URDME 1.3 release in short

The major changes compared to URDME 1.2 [2] are as follows:

1. The syntax and behavior of the `urdme`-interface has been straightened out and simplified considerably. Type `help urdme` for more information, or `help nsm` for information on the default NSM-solver.

2. Two new utility functions, `rparse` and `rparse_inline`, allow for automatic construct of C-source code propensities or, alternatively, so-called *"inline propensities"*, and the associated stoichiometric data. Using either of these dramatically changes the way one works as it allows for defining models 'on the fly', never explicitly editing C-language propensities.

3. URDME 1.3 includes support for Comsol Multiphysics 5.x as well as for PDE Toolbox.

4. There are three new solvers: AEM, SSA, and UDS. The DFSP solver from 1.2 is no longer included.

5. URDME now relies on the portable `mex`-command rather than on make-files for its compilation. Solver data is always passed in memory; passing arguments via files is no longer enforced.

6. Propensity functions now include a new argument `gdata`, useful for passing global arguments like rate constants without re-compilation. Also, the interface to allocating propensity functions has been changed and now allows for safety checks.

7. Propensities for mass-action models can as before be defined directly in the Matlab layer. URDME 1.3 relies on the inline propensities defined in URDME 1.0 rather than on the compiled versions of URDME 1.2.

8. The SBML-interface has not been maintained with URDME 1.3, but is considered for a subsequent release.

Downloading/installing There is no install procedure, simply download URDME 1.3 and after calling the `startup`-function for setting the paths you are ready to simulate.

System requirements Please refer to the file VERSION, or consult the web-page. Briefly: Matlab version 8 or higher, Comsol 5, PDE Toolbox version 1.5 or higher.

License URDME is work in progress. You may use, distribute, and modify the code freely under the GNU GPL license version 3. We welcome contributions, suggestions, comments, and bug-reports. Refer to the file LICENSE for a full statement.

Quick start Navigate to the examples folder and pick one of the examples. Some of the examples require a live Comsol connection.

Web-page `http://www.urdme.org`.

3 Software overview

URMDE consists of three logical layers. The top layer is made up of an interface to an external PDE solver and pre-/post-processing engine, currently Comsol Multiphysics or PDE Toolbox. The use of this layer is to define diffusion- or transport rates. The bottom layer is a set of simulation routines written in a compiled language (typically C). Interfacing those two levels is a middle layer in the Matlab environment, designed to facilitate data processing and visualization, as well as custom model development. Together these layers form a software package that enables powerful development and efficient simulation of complex models of spatial stochastic phenomena.

The URDME structure is designed with both efficiency and flexibility in mind. In Matlab, an URDME model is defined by a single entity, *the URDME structure*, conventionally called `umod`. This structure carries all information about the model to be simulated. Indeed, the most bare use of `urdme` is

```
>> umod = urdme(umod);
```

Depending on the contents of `umod`, this call typically compiles the propensity source file defined in the field `umod.propensities` using the `mex`-based compilation script named `make_⟨umod.solver⟩.m`. After compilation the solver `mex⟨umod.solver⟩` is called with arguments formed from the fields of `umod` and the result of the simulation is attached to the field `umod.U`. A more explicit call achieving the same thing is

```
>> umod = urdme(umod,'propensities',<file>,'solver',<solver>);
```

Type `help urdme` for more information on the different options available.

A model is conveniently built in three separate steps, one for each of the logical layers. For example, the geometry of the model can be defined in a Comsol `.mph` model file, along with the names and diffusion rates of each chemical species. A Matlab model file supplies the model with the stoichiometric matrix, the dependency graph, and the initial state of the system. Briefly, the stoichiometric matrix defines the effect of the chemical reactions on the state of the system while the dependency graph indicates the reaction rates that need to be updated after a given reaction or diffusion event has occurred. Finally, a model file written in a compiled language specifies the propensity functions for the chemical reactions in the system. Using compiled rather than interpreted reaction rates ensures maximum efficiency when simulating the model. Alternatively, for mass-balance kinetics, the very efficient inline propensities may be used instead.

3.1 The modeling steps in some more detail

The steps involved in performing a URDME simulation is outlined below, along with the routines that perform the different tasks.

1. Process the .mph model file. This is achieved by loading the Comsol Java object into the Matlab workspace and invoking the routine `comsol2urdme`. This initializes the `umod`-structure with various fields and additionally stores the original Comsol Java object in the field `umod.comsol`. The `umod`-structure contains the fields `D`, `vol`, and `sd`, i.e. those data structures related to the geometry of the model and to the unstructured mesh. See Table 3.2.

   As a somewhat less advanced alternative, PDE Toolbox may be used instead of Comsol for this step. See `pde2urdme`.

2. The next step is to use Matlab to initialize the remaining essential data fields, `tspan`, `u0`, `N`, and `G`. Optional fields include `ldata`, `gdata`, and `solverargs`. They should all be added as fields to `umod`. Any modifications of the data structures added to the model by `comsol2urdme` in the previous step is typically performed in this step as well. Again, see Table 3.2.

   The reaction propensities need to be specified, either as data matrices in a certain format ("inline propensities"), or as source code to be compiled. Consider using the utility functions `rparse` or `rparse_inline` at this stage.

3. After `umod` is complete, `urdme` is called. Some additional arguments may be parsed directly to `urdme` as property/values. `urdme` will call the function `urdme_validate` to perform error-checking on the input to make sure that all required fields in `umod` are present and have the correct properties. Finally, the chosen solver and propensities are compiled using `mex` and then called by `urdme`.

4. After successful simulation, the resulting trajectory is written to the field `umod.U`. This can then be transferred back to Comsol for visualization via the function `urdme2comsol` (for an alternative, see `urdme2pde`).

Table 3.1 shows the directory structure of URDME together with a short description of each routine. Table 3.2 similarly lists the fields of the URDME structure.

4 Details and specifications

In this section we give a detailed description of the input to the URDME solvers.

4.1 The mex-interface

The URDME solver sequence is readily summarized as:

```
>> mexmake_<umod.solver>(umod.propensities);
>> umod.U = mex<umod.solver>(umod.tspan,umod.u0, ...
                             umod.D,umod.N,umod.G, ...
                             umod.vol,umod.ldata,umod.gdata,umod.sd, ...
                             umod.report,umod.seed, ...
                             umod.solverargs);
```

For the meaning of the different fields in `umod`, see Table 3.2. Samples of actual source code handling the final `mex`-interface call are found in the source folders. Contributed solvers should adhere to exactly the above `mex`-interface.

4.2 Specifying propensities for chemical reactions

We have provided two separate methods to specify the reaction propensities. Simple polynomial rate laws (mass-action) can be provided as inline propensities and can be specified in

Directory	File(s)	Description
comsol	comsol2urdme.m	Matlab function converting Comsol's Java object to a valid `urdme`-struct `umod`.
	urdme2comsol.m	Matlab function for conversion of the output of `urdme` to the solution format used by Comsol.
pde	pde2urdme.m<br>urdme2pde.m	PDE Toolbox interface routines, as above.
doc	manual.pdf	The most recent version of this manual.
include	binheap.h	Binary heap for managing events in some solvers.
	inline.h	Declaration of the inline propensity function.
	propensities.h	Definition of the propensity function datatype.
	report.h	Header for report.c.
msrc	urdme.m	Main solver routine.
	urdme_validate.m	Input validation.
msrc/utils	rparse.m	Propensity C-code generation.
	rparse_inline.m	Inline propensity data generation.
src	binheap.c	Implementation of the binary heap.
	inline.c	Inline propensity function definition.
	propensities.c	Empty, used for empty propensity source.
	report.c	Default URDME reporter.
src/nsm		Included solvers: NSM [8] is the default solver.
src/aem		All events method [3].
src/uds		URDME Deterministic solver [10].
src/ssa		For help on one solver, type e.g., `help ssa`.
examples	(various)	See Section 5.

Table 3.1: Overview of the files and routines that make up URDME.

Name	Type	Description
tspan	Vector	A sequence of points in time where the state of the system is to be returned.
u0	Matrix [Mspecies×Ncells]	u0(i, j) is the initial number of species i in subvolume j.
D	Sparse matrix [Ndofs× Ndofs], where the total number of degrees of freedom is Ndofs = Mspecies× Ncells	The *transpose* of the diffusion matrix $M^{-1}K$ obtained from the FEM discretization of the macroscopic diffusion equation, cf. (A.5). Each column in D (i.e. each row in $M^{-1}K$) corresponds to a subvolume, and the non-zero coefficient $D(i, j)$ gives the diffusion rate constant from subvolume i to subvolume j.
N	Sparse matrix [Mspecies × Mreactions]	The stoichiometric matrix. Each column corresponds to a reaction, and execution of reaction j amounts to adding the jth column to the state vector.
G	Sparse matrix [Mreactions × (Mspecies+Mreactions)]	Dependency graph. The first Mspecies columns correspond to diffusion events and the following Mreactions columns to reactions. A non-zeros entry in element i of column j indicates that propensity i needs to be updated if the event j occurs. See Section 5 for examples.
vol	Vector [Ncells]	The volume of the macroelements, i.e. the diagonal elements of the lumped mass-matrix M (cf. Appendix A.2).
sd	Vector [Ncells]	The subdomain numbers of all subvolumes. See Section 5 for more details.
solver	String	Name of solver.
propensities	String	Propensity source file.
report	Scalar	Report level, typically 0, 1, or 2.
compile	Boolean	Compilation on/off.
parse	Boolean	Parsing on/off.
seed	Scalar	Random seed value.
ldata	Matrix [dsize×Ncells]	Local data vector. A pointer to column j is passed as an additional argument to the propensities in subvolume j.
gdata	Vector [⟨anything⟩]	Global data vector
solverargs	Cell-vector of property/value pairs.	Solver arguments, must be parsed in this form by the solver mex-interface.
U	Matrix [Ncells× length(tspan)]	Latest stored solution.
comsol, pde	Object fields	Comsol Java object and PDE Toolbox data.
private	Object field	Arbitrary additional data.

Table 3.2: The fields of the URDME structure. *Top:* required before call to urdme, *2nd from top:* passed as arguments to urdme with default values, *3rd:* optional fields with empty defaults, *bottom:* optional fields.

the Matlab layer. For general propensities and full flexibility, the rate laws can be specified in a model file written in a compiled language (C typically).

Note that one can easily use *both* inline and compiled propensities simultaneously. This might be convenient when only a few propensities are complicated and has to be compiled.

4.2.1 Inline propensities

An “inline propensity” is a compact data format for specifying basic chemical reactions with polynomial rate laws. An inline propensity P_r can be defined as

$$P_r(x) = \begin{cases} \frac{k_1 x_i x_j}{\Omega} + k_2 x_k + k_3 \Omega & \text{if } i \neq j, \\ \frac{k_1 x_i (x_i - 1)}{2\Omega} + k_2 x_k + k_3 \Omega & \text{if } i = j. \end{cases}$$

Here x is the column in `x` which contains the state of the subvolume considered and Ω is the corresponding volume. The coefficients and indices are specified in matrices `K` and `I` where $\texttt{K}(:, r) = [k_1\, k_2\, k_3]^T$ and $\texttt{I}(:, r) = [i\, j\, k]^T$ are the constants corresponding to the rth inline propensity. The matrix `S` is a (possibly empty) sparse matrix such that $\texttt{S}(:, r)$ lists all subdomains in which the rth inline propensity is turned off. *Note that no inline propensities are active in subdomain zero!* A complete example of the use of inline propensities can be found in the 'annihilation' example folder.

! *The format specification for inline propensities might feel a bit complicated at first!* The utility function `rparse_inline` can aid in constructing these from simple expressions.

4.2.2 Compiled propensities

The other way to specify propensity functions is to supply them to `urdme` as a propensity file written in C. The precise form of the propensity functions is defined by the data type `PropensityFun`, defined in the header 'propensities.h' (found in the 'include' directory) as

```
typedef double (*PropensityFun)(const int *x,double t,double vol,
                                const double *ldata,const double *gdata,
                                int sd);
```

The arguments `vol`, `ldata`, `gdata`, and `sd` are described in Table 3.2. Additionally, the input vector `x` of length `Mspecies` is the copy number in a given subvolume, and `t` is the absolute time. Note that, of the current URDME solvers, only UDS makes an active use of the time.

Below is a commented example of a model file defining a simple chemical system composed of a single species undergoing a dimerization reaction.

```
/* Propensity definition of a simple dimerization reaction. */
#include "propensities.h"
#include "report.h"

const int NR = 1; /* number of reactions */

const double k = 1.0e-3; /* rate constant */

/* forward declaration */
double rFun1(const int *x,double t,double vol,
             const double *ldata,const double *gdata,int sd);

/* static propensity vector */
static PropensityFun ptr[] = {rFun1};
```

```
double rFun1(const int *x,double t,double vol,
             const double *ldata,const double *gdata,int sd)
/*  X + X --> 0. */
{
  return k*x[0]*(x[0]-1)/vol;
}

PropensityFun *ALLOC_propensities(size_t Mreactions)
{
  if (Mreactions > 1) PERROR("Wrong number of reactions.");
  return ptr;
}

void FREE_propensities(PropensityFun *ptr)
{ /* do nothing since a static array was used */ }
```

A propensity file *must* implement the following routines:

- `PropensityFun *ALLOC_propensities(size_t Mspecies)`
- `void FREE_propensities(PropensityFun *ptr)`

The first function should allocate and initialize an array of function pointers to the propensity functions and return a pointer to this array. This is the function that the solvers will call to access the rate functions. The second function should deallocate the pointer `ptr`, whenever this is required. In the above example, a static array was used and deallocation is unnecessary. For further examples, see Section 5.

! *The propensity function specification can be a bit cumbersome at first!* Consider starting your work with the function `rparse`. For example, the above example is readily generated directly as

```
>> rparse({'X+X > k*X*(X-1)/vol > @'},{'X'},{'k' 1e-3});
```

Note that the fields `umod.N` and `umod.G` can be generated as well.

5 Worked examples

In this section we describe the general workflow involved in setting up and simulating a model in URDME using the Comsol and Matlab interfaces. The major steps are (compare Section 3.1):

1. *Specify the model.* This involves defining the geometry, mesh, initial conditions and chemical reactions of the model. In URDME, this is conveniently accomplished using three model files: a Comsol model file 'model.mph', a Matlab model file 'model.m' and a reaction propensity file 'model.c', where we use *model* as a placeholder for the non-extension part of the file-name.

   (a) The Comsol model defines the geometry of the problem and Comsol Multiphysics is used to create a mesh representing the spatial discretization of the diffusion equation with Neumann boundary conditions and the inter-voxel diffusion jump coefficients. The Comsol model is exported to the Matlab layer, typically using the `mphload` command. The function `comsol2urdme` then extracts the information into a startling `umod`-structure. A simpler alternative to Comsol is PDE Toolbox. Below we show examples of both methods.

(b) The Matlab model file specifies the chemical reaction networks of the problem.

(c) The propensity functions for the chemical reactions are either specified in a C-source code file and/or in the Matlab layer as inline propensities. These are typically created using the utility functions `rparse` and/or `rparse_inline`.

2. *Run the simulation.* The simulation takes place in the Matlab workspace via a call to `urdme`.

3. *Post-processing.* After a normal termination of the solver, a trajectory of the stochastic process will be attached to the field `umod.U`. The function `urdme2comsol` attaches this trajectory to the Comsol Java object in `umod.comsol`. At this point, you can use all post-processing options available in the Comsol interface to visualize the results. You may also save the solution in `umod.comsol` to file via the command `mphsave` and later post-process it in the Comsol GUI. Again and as an alternative, you may rely on PDE Toolbox rather than on Comsol.

5.1 Morphogenesis: the Schnakenberg model

As an immediate example of the simulation capabilities and general workflow of URDME, we reproduce one of the simulations found in [30], namely a stochastic version of the Schnakenberg model. The model consists of two *morphogens* U and V diffusing in some geometry and reacting according to the transitions

$$\begin{array}{ccc} \emptyset & \xrightarrow{k_1\Omega} & U \\ U & \xrightarrow{k_2 U} & \emptyset \\ \emptyset & \xrightarrow{k_3\Omega} & V \\ U+U+V & \xrightarrow{k_4/\Omega^2\,U(U-1)V} & U+U+U \end{array} \tag{5.1}$$

We set up and solve the model step by step as follows; see also the file `morphogenesis2D_run.m` in the folder 'examples/morphogenesis'.

Defining the geometry and diffusion rates in PDE Toolbox We start by building the geometry using the format supported by PDE Toolbox, in this case a 2D torus:

```
C1 = [1 0 0 50]';
C2 = [1 0 0 15]';
gd = [C1 C2];
sf = 'C1-C2';
ns = char('C1','C2')';
G = decsg(gd,sf,ns);
```

We next build the mesh as follows:

```
[P,E,T] = initmesh(G,'hmax',2.5);
pdemesh(P,E,T), axis tight, axis equal
```

The result of the plot-command is displayed in Figure 5.1.

The diffusion parameter is different for the two species. We use PDE Toolbox to assemble the discretized diffusion operator:

```
D_U = 1;
D_V = 40;
umod = pde2urdme(P,T,{D_U D_V});
umod.sd = ceil(umod.sd); % (not used)
```

PDE Toolbox produces an interpolated value of `sd` and a 'decision' is required for how to handle this value. Above we simply round the result upwards; a model making more advanced use of the subdomain numbering needs to handle this issue with more care.

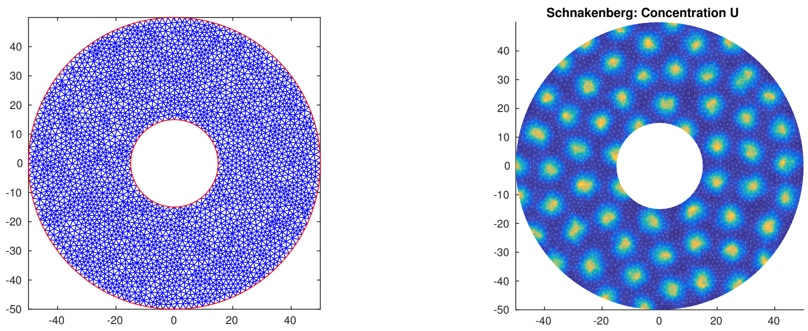

Figure 5.1: Visualizations from the Schnakenberg model.

Specifying the chemical reactions We readily construct the propensity file using the utility-function `rparse`:

```
r1 = '@ > k1*vol > U';
r2 = 'U > k2*U > @';
r3 = '@ > k3*vol > V';
r4 = 'U+U+V > (k4/(vol*vol))*(U*(U-1)*V) > U+U+U';
[~,umod.N,umod.G] = rparse({r1 r2 r3 r4},{'U' 'V'}, ...
                            {'k1' 0.1 'k2' 1 'k3' 0.9 'k4' 1}, ...
                            'schnakenberg.c');
```

The result of the above command is written on the indicated file. Take a moment to study the resulting source code!

We also need to define a few more fields of the `umod`-struct:

```
umod.u0 = zeros(size(umod.N,1),numel(umod.vol));
umod.tspan = 0:10:100;
umod.vol = 50/mean(umod.vol)*umod.vol;
```

The last command is special to the current model: it is of some interest to investigate how the Schnakenberg model responds to different scaling of the system volume. Here this is achieved by directly rescaling the field `vol` of the `umod`-struct.

Simulating the model At this stage we simply invoke `urdme`:

```
umod = urdme(umod,'propensities','schnakenberg','report',3);
```

! Using `report` = 3 implies that you have the possibility to discontinue the simulation at each entry in `tspan`. A useful feature when setting up new models.

Postprocessing To use PDE Toolbox in the visualization process we rely on the function `urdme2pde`:

```
umod = urdme2pde(umod);
figure, clf,
pdesurf(umod.pde.P,umod.pde.T,umod.pde.U(1,:,end)');
title('Schnakenberg: Concentration U');
view(0,90), axis tight, axis square, colormap('parula')
```

Again, the result of the plot-command is displayed in Figure 5.1. In the file `morphogenesis2D_run.m` a second model, *the Brusselator*, is also solved in a similar fashion.

5.2 Min oscillations in *E. Coli*

To illustrate the solution steps in a more advanced 3D model, we will reproduce simulations of the Min-system from [16]. The geometry will be a model of an *E. coli* bacterium. It is rod-shaped with length $3.5\mu m$ and diameter $0.5\mu m$. The reactions and parameters of the model can be found in Table 5.1. The model is built in the file `mincde_run.m` found in the folder 'examples/mincde'.

$\text{MinDcytATP} \xrightarrow{k_d} \text{MinDmem}$	$\text{MinDcytATP} + \text{MinDmem} \xrightarrow{k_{dD}} 2\text{MinDmem}$
$\text{MinE+MinDmem} \xrightarrow{k_{de}} \text{MinDE}$	$\text{MinDE} \xrightarrow{k_e} \text{MinDcytADP} + \text{MinE}$
$\text{MinDcytADP} \xrightarrow{k_p} \text{MinDcytATP}$	

Table 5.1: The chemical reactions of the Min system. The constants take the values $k_d = 0.0125\mu m^{-1}s^{-1}$, $k_{dD} = 9 \times 10^6 M^{-1}s^{-1}$, $k_{de} = 5.56 \times 10^7 M^{-1}s^{-1}$, $k_e = 0.7s^{-1}$, and $k_p = 0.5s^{-1}$.

5.2.1 Setting up the model for simulation

Defining the geometry and diffusion rates in Comsol Multiphysics

1. Open Comsol and use the Model Wizard to create the model template. Select '3D' as space dimension and add the physics module 'Chemical Species Transport / Transport of Diluted Species' in the next step. In the 'Dependent variables' window chose the 'Number of species' to be `5` and in the 'Concentrations' list enter the names `MinDcytATP, MinDmem, MinE, MinDE` and `MinDcytADP`. You may also enter these variable names at a later stage, see below. Select the 'Time Dependent' study type in the next step of the wizard and click on the flag symbol to create the template.

   ! *Note that the 'Chemical engineering module' is not required in general for URDME, but is used in this example for convenience.*

2. Next we create the geometry. We will build the rod shaped domain from two spheres and one cylinder. Right click on 'Geometry 1', select the 'Cylinder' option and in the radius and height field enter `0.5e-6` and `3.5e-6`. Click on the 'Build Selected' Button and you should now see a cylinder in your workspace. Now, select the 'Sphere' node from the 'Geometry' context-menu and and enter `0.5e-6` in the radius field. Create another identical sphere but enter `3.5e-6` as the x-coordinate. Click on 'Build All' and observe the created domain in the graphics-window.

   Right click on 'Geometry' again and select 'Boolean Operations > Union'. Select all three domains and add them to the 'Input objects' selection. Uncheck the 'Keep interior boundaries' box and complete the geometry creation by pushing the 'Build All' button. The final geometry has 1 domain, 12 boundaries, 20 edges, and 10 vertices.

3. Having constructed the geometry, the next step is to specify the parameters in the model. If you haven't specified the variable names yet, do this now under 'Transport of Diluted Species > Dependent Variables' (Number of species: 5, Concentrations: `MinDcytATP`, `MinDmem`, `MinE`, `MinDE`, `MinDcytADP`).

   In the physics settings 'Transport of Diluted Species > Transport Mechanisms', deactivate the flag on 'Convection'. Also, with the 'Transport of Diluted Species' tab active, press the advanced property icon (an all-seeing eye just under the 'Model Builder' title), and select 'Discretization'. Use linear elements and uncheck the 'Compute boundary fluxes'.

   Next we need to specify the diffusion constants of the species in the 'Diffusion' node of the physics menu 'Transport Properties'. Enter the diffusion coefficients `2.5e-12` for

`MinDcytATP`, `MinE`, and `MinDcytADP`. For `MinDE` and `MinDmem` the diffusion constant should be `1e-14`. The units of all constants are m^2/s.

! *`MinDE` and `MinDmem` are species bound to the membrane, hence their lower diffusion rates. We have not specified this explicitly at this stage, but will do so later in the Matlab layer.*

4. In order to be able to distinguish between the interior of the bacterium and the membrane, we must also create two domains which URDME can parse. One interior domain that represents the cytoplasm and one boundary domain that represents the membrane. This is done by (1) defining the global `urdme_sdlevel` variable, and (2) defining the variable `urdme_sd` on the geometry as an expression with different values in the different subdomains. The latter variable is then used by URDME to distinguish the nodes on the boundary from those in the interior. Somewhat technically, the former variable, `urdme_sdlevel`, is required to correctly evaluate the value of `urdme_sd` on the different manifolds of the model (points, edges, boundaries, domain).

   First click right on 'Global Definitions' and create the 'Variable' `urdme_sdlevel`. Assign this variable the value 2 to indicate that the *lowest* dimension where `urdme_sd` is defined is on the boundaries of the geometry (where the dimensionality is 2).

   Second, click right on the menu 'Definitions', and create two 'Variables'. Label the first one `urdme_sd_dom` (name is not critical) and select the 'Geometry entity level' to be 'Domain' and chose the 'Selection' to be 'All domains'. Now, enter a new variable in the window below by specifying the name to `urdme_sd` and expression to `1`. Similarly, label the second variable `urdme_sd_bnd` (again, name is not critical), specify the geometric entity level to 'Boundary' and set the 'Selection' to 'All boundaries'. Enter the variable name `urdme_sd` into the 'Variables' window and set the expression to `2`.

   All in all we have now defined the variable `urdme_sdlevel` to be 2 globally such that URDME will correctly evaluate the variable `urdme_sd` first at the entire domain of the model (where `urdme_sd` = 1) and second at the boundaries of the model (where `urdme_sd` = 2). The URDME convention here is that lower dimensional manifolds take precedence over higher dimensional ones.

5. In the 'Mesh' node, set 'User controlled mesh' as sequence type and in the appeared 'Size' node select the 'Custom' option. Set the maximum element size to `1e-7` and press 'Build All'. Now click on the 'Study' node and press the 'Compute' button.

6. Some Comsol callback functions require a placeholder solution. To create a simple such solution, under 'Study 1: Time Dependent', select 'Times: range(0,1,1)' and press the 'Compute' button.

Now you need to transfer the created model into Matlab. Make sure that you are connected to the Server, if not, connect via 'File > Client Server > Connect to Server'. When having a working connection the export can be performed by selecting 'File > Client Server > Export Model to Server'.

Another option is to save the model to file (here: `coli.mph`), and open it later in a running Matlab session with 'LiveLink' via the command `mphload`:

```
model = mphload('coli.mph'); % load Comsol model
umod = comsol2urdme(model);  % create URDME struct
```

Specifying the chemical reactions The chemical reactions are specified in a separate file written in C, conveniently generated on the fly by the utility function `rparse`. Open the file 'examples/mincde/mincde.m'. We will walk through the contents of this file and explain

what the different parts do. Additional information can also be found in the comments in the file.

First out is the membrane bound reaction,

```
r1 = 'MinDcytATP > sd == 2 ? kd*MinDcytATP/ldata[0] : 0.0 > MinDmem';
```

Note how `sd` is used to check if the voxel belongs to the membrane or not. We have to make sure, however, that we keep track of what value we assigned to the different subdomains in the Comsol model file (the value of the expression `urdme_sd`).

Note also how the first reaction in the model contains a scaling with the local length scale of the subvolume. For a uniform Cartesian mesh this would simply have been the (constant) side lengths of the cubes in the mesh. For the unstructured mesh however, this value will be different in every subvolume. It is readily obtained from Comsol, and is passed to the propensity function via the data vector `ldata` which will be initialized with the correct values below.

We continue with the two bimolecular reactions,

```
r2 = ['MinDcytATP + MinDmem > kdD*MinDcytATP*MinDmem/(1000.0*NA*vol)' ...
      '> MinDmem+MinDmem'];
r3 = 'MinE + MinDmem > kde*MinE*MinDmem/(1000.0*NA*vol) > MinDE';
```

Note the unit conversions given explicitly in the bimolecular propensity function. The rate constants for the bimolecular reactions in this model are given in the unit $M^{-1}s^{-1}$ and need to be converted to mesoscopic rates. That is why we divide with Avogadros number times the volume of the subvolume. Also, the way we have set up the geometry model file, the volume is given in the unit m^3, and needs to be converted to L^3. URDME cannot keep track of matching the units between the different model files automatically: this is the responsibility of the end-user.

The chemical network is concluded with two degradation reactions:

```
r4 = 'MinDE > ke*MinDE > MinDcytADP + MinE';
r5 = 'MinDcytADP > kp*MinDcytADP > MinDcytATP';
```

To create the propensity file we define the species and the rate constants and then invoke `rparse`:

```
species = {'MinDcytATP' 'MinDmem' 'MinE' 'MinDE' 'MinDcytADP'};
rates = {'NA' 6.022e23 'kd' 1.25e-8 'kdD' 9.0e6 'kde' 5.56e7 ...
         'ke' 0.7 'kp' 0.5};
[F,umod.N,umod.G] = rparse({r1 r2 r3 r4 r5},species,rates,'fange.c');
```

The propensity C-file is now found in the file `fange.c`. Take a moment to study this file! The code itself is also returned in the output Matlab character array `F`.

Before we can run the simulation, we need to modify the diffusion rates that we obtain from the initial Comsol model so that the membrane-bound species only diffuse on the membrane. We have already prepared for this by labeling the subvolumes next to the boundary using the expression `urdme_sd` in the Comsol model.

1. *The initial condition.* There is complete freedom in specifying the initial condition. In the present case we simply distribute 4002 `MinDcytATP` and 1040 `MinE` molecules in some random way in the entire bacterium.

   ```
   % the total number of molecules of the species
   nMinD = 4002;
   nMinE = 1040;

   % assign randomly
   ```

```
u0 = zeros(Mspecies,Ncells);
ind = floor(Ncells*rand(1,nMinE))+1;
u0(3,:) = full(sparse(1,ind,1,1,Ncells));
ind = floor(Ncells*rand(1,nMinD))+1;
u0(5,:) = full(sparse(1,ind,1,1,Ncells));
umod.u0 = u0;
```

Note that the code above does not produce a uniformly random initial distribution since the volume of each voxel is not taken into account.

2. *Specifying the times to output the state of the system.* URDME will look for a vector `tspan` to determine when to output the state of the trajectory (the number of events generated in a typical realization often exceeds 10^9 so we can't output after each event). Here, we want to sample the system on the time interval $[0, 200]$ seconds, with output each second. This is achieved by

```
umod.tspan = 0:200.
```

3. *Membrane diffusion.* In order to make `MinDmem` and `MinDE` diffuse only on the membrane, we will zero out all elements in the diffusion matrix that are in the cytosol. To obtain indices of those subvolumes we use the information in the subdomain vector `sd`.

```
cyt = find(umod.sd == 1);
pm  = find(umod.sd == 2);
```

Remember that we gave `urdme_sd` the value 2 on the membrane and 1 in the interior. The diffusion matrix `D` will contain the rate constants for the diffusive events on the unstructured mesh. `D` is generated by Comsol and is available in the field `umod.D`. To (efficiently) zero out the correct entries in `D`, we first decompose the sparse matrix, find the entries using `pm` and `cyt` above, and then reassemble the matrix again (compensating for the removed entries by adjusting the diagonal of the matrix). All in all, the code to do this is as follows:

```
% For MinDmem (2) and MinDE (4), flag all dofs in the cytosol for
% removal.
ixremove = [];
for s = [2 4]
  ixremove = [ixremove; Mspecies*(cyt-1)+s];
end

% Decompose the sparse matrix.
D = umod.D';
[i,j,s] = find(D);

% Set all elements in the diffusion matrix corresponding
% to the cytosol to zero.
ixremove = [find(ismember(i,ixremove)); find(ismember(j,ixremove))];
i(ixremove) = [];
j(ixremove) = [];
s(ixremove) = [];

% Reassemble the sparse matrix and adjust the diagonal entries.
ixkeep = find(s > 0);
```

```
D = sparse(i(ixkeep),j(ixkeep),s(ixkeep),Ndofs,Ndofs);
d = full(sum(D,2));
D = D+sparse(1:Ndofs,1:Ndofs,-d);
umod.D = D';
```

! It is of fundamental importance that the columns of `D` sum to zero, and that all off-diagonal entries are positive. For an introduction to how `D` is constructed, see Appendix A. For a detailed account, consult [14].

! The way we have modeled membrane diffusion is simply by saying that the subvolumes closest to the membrane constitute the membrane layer. As the mesh becomes finer near the boundary, the thickness of this layer will decrease, eventually approaching a 2D model of the membrane. One can also think of other ways of modeling the membrane diffusion.

4. *The local data vector.* Finally, we need to set `umod.ldata` to contain the values of the length parameter for the subvolumes (it is needed in the first reaction). To do this, we use the built-in Comsol function `mphinterp` which can be used to evaluate an expression in any point in the domain. Here, we simply get the subvolume sizes by using the pre-defined expression h, evaluated in the vertices of the mesh.

   Relying on Comsol Multiphysics:

```
xmi = mphxmeshinfo(umod.comsol);
umod.ldata = mphinterp(umod.comsol,'h','coord', ...
                       xmi.dofs.coords(:,1:Mspecies:end),'solnum',1);
umod.ldata = umod.ldata(xmi.dofs.nodes(1:Mspecies:end)+1);
```

! For more details concerning the internal ordering of the dofs, consult the Comsol user's manual. The interface routines `comsol2urdme` and `urdme2comsol` also contain useful information on this matter.

5.2.2 Running the simulation

With the model set up correctly, we are now ready to simulate:

```
umod = urdme(umod,'propensities','fange','report',2);
```

URDME will now compile the solver with linking to the propensities specified in `fange.c`, and then execute the solver. The result of the simulation is stored in `umod.U`. To prepare for post-processing we can transfer this result back to the Comsol object,

```
umod = urdme2comsol(umod);
```

5.2.3 Post-processing

If the simulation in the previous step completed without errors, the model structure will now contain a realization of the stochastic process. To visualize the trajectory, we can use any of the visualization options available in Comsol or we can create routines of our own. To look at the `MinDmem` distribution on the membrane at the final time we can use Comsol's post-processing functionality.

This command creates a plot-container for the visualization to follow:

```
umod.comsol.result.create('res1','PlotGroup3D');
```

To visualize the result at a specific time, e.g., after 100s:

```
umod.comsol.result('res1').set('t','100');
```

! *You can specify any time in the interval you simulated, but if you specify a time that lies between two points in* `tspan` *Comsol will do interpolation to approximate the result at that point.*

To visualize the result of the simulation on the surface we can use:

```
umod.comsol.result('res1').feature.create('surf1','Surface');
umod.comsol.result('res1').feature('surf1').set('expr','MinDmem');
mphplot(umod.comsol,'res1');
```

Where we can replace the string `'MinDmem'` with the name of any other species.

To visualize the solution inside the domain, we need to first create a new plot container.

```
umod.comsol.result.create('res2','PlotGroup3D');
umod.comsol.result('res2').set('t','100');
```

Now we can visualize the solution on a 'slice' of the zx-axis of the model.

```
umod.comsol.result('res2').feature.create('slc2','Slice');
umod.comsol.result('res2').feature('slc2').set('expr','MinDcytATP');
umod.comsol.result('res2').feature('slc2').set('quickplane','zx');
umod.comsol.result('res2').feature('slc2').set('quickynumber','1');
mphplot(model,'res2');
```

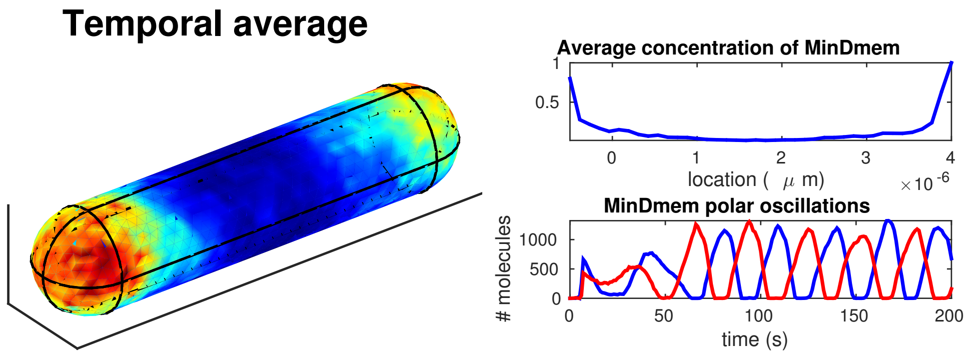

Figure 5.2: Visualizations from the Min system.

There are many more options that can be passed to `mphplot` to control the plot produced. For a detailed account, see the Comsol documentation:

```
>> help mphplot
```

If you prefer to work within the Comsol GUI for visualization, you can import back the Comsol model with the attached stochastic trajectory into Comsol. This can be done by typing:

```
>> mphsave(umod.comsol,'<output_filename>.mph')
```

Optionally, from the Comsol GUI, import the new structure (`umod.comsol`): 'File > Client Server > Import Model from Server'. You can now visualize the trajectory using the options provided in the 'Results' node.

6 Integrating solvers with URDME

URDME is easily enriched with additional third party solvers. URDME plugins have three main components: the `mex`-based makefile, the `mex`-interface source code, and (conventionally) a placeholder Matlab .m-file with defining help-text. Each part is described in Table 6.1 where the files that make up the NSM solver are explained. We recommend that developers follow this format when integrating their own solvers.

File	Description
mexmake_nsm.m	A `mex`-based makefile for building the solver. The name of this file is important: the automatic compilation process looks for a makefile that is suffixed with the name of the solver (in lower case). This makefile compiles the solver with the model's propensity functions into the executable `mexnsm` which is called by the Matlab-level interface.
mexnsm.c	Solver entry point, a `mexFunction`.
nsm.h, nsm.c	Actual low-level source code implementing the NSM solver.
nsm.m	Empty Matlab .m-file containing help-text.

Table 6.1: Overview of the files that make up the NSM solver.

Acknowledgment

The development of URDME 1.3 was partially funded by the Linnaeus center of excellence UPMARC, Uppsala Programming for Multicore Architectures Research Center.

References

[1] Naama Barkai and Stanislav Leibler. Circadian clocks limited by noise. *Nature*, 403:267–268, 2000.

[2] P. Bauer, B. Drawert, S. Engblom, and A. Hellander. URDME v. 1.2: User's manual. Technical Report 2012-036, Dept of Information Technology, Uppsala University, 2012. Available at http://arxiv.org/abs/0902.2912.

[3] P. Bauer and S. Engblom. Sensitivity estimation and inverse problems in spatial stochastic models of chemical kinetics. In A. Abdulle, S. Deparis, D. Kressner, F. Nobile, and M. Picasso, editors, *Numerical Mathematics and Advanced Applications: ENUMATH 2013*, volume 103 of *Lecture Notes in Computational Science and Engineering*, pages 519–527, Berlin, 2015. Springer.

[4] Yang Cao, Dan T. Gillespie, and Linda Petzold. Multiscale stochastic simulation algorithm with partial equilibrium assumption for chemically reacting systems. *J. Comput. Phys.*, 206:395–411, 2005.

[5] Comsol Inc. *Comsol Multiphysics Reference Guide*, 5.2 edition, 2017.

[6] B. Drawert, S. Engblom, and A. Hellander. URDME: a modular framework for stochastic simulation of reaction-transport processes in complex geometries. *BMC Syst. Biol.*, 6(76):1–17, 2012.

[7] Weinan E, Di Liu, and Eric Vanden-Eijnden. Nested stochastic simulation algorithm for chemical kinetic systems with disparate rates. *J. Chem. Phys.*, 123, 194107, 2005.

[8] Johan Elf and Måns Ehrenberg. Spontaneous separation of bi–stable biochemical systems into spatial domains of opposite phases. *Syst. Biol.*, 1(2), 2004.

[9] Michael B. Elowitz, Arnold J. Levine, Eric D. Siggia, and Peter S. Swain. Stochastic gene expression in a single cell. *Science*, 297(5584):1183–1186, 2002.

[10] S. Engblom. Computing the moments of high dimensional solutions of the master equation. *Appl. Math. Comput.*, 180(2):498–515, 2006.

[11] Stefan Engblom. *Numerical Solution Methods in Stochastic Chemical Kinetics*. PhD thesis, Uppsala University, 2008.

[12] Stefan Engblom. Galerkin spectral method applied to the chemical master equation. *Commun. Comput. Phys.*, 5(5):871–896, 2009.

[13] Stefan Engblom. Spectral approximation of solutions to the chemical master equation. *J. Comput. Appl. Math.*, 229(1), 2009.

[14] Stefan Engblom, Lars Ferm, Andreas Hellander, and Per Lötstedt. Simulation of stochastic reaction–diffusion processes on unstructured meshes. *SIAM J. Scientific. Comp.*, 31(3):1774–1797, 2009.

[15] Stewart N. Ethier and Thomas G. Kurtz. *Markov Processes: Characterization and Convergence*. Wiley series in Probability and Mathematical Statistics. John Wiley & Sons, New York, 1986.

[16] David Fange and Johan Elf. Noise-induced Min phenotypes in *E. coli*. *PLOSB*, 2(6):0637–0647, 2006.

[17] Lars Ferm and Per Lötstedt. Adaptive solution of the master equation in low dimensions. *Appl. Numer. Math.*, 59(1):265–284, 2009.

[18] Michael A. Gibson and Jehoshua Bruck. Efficient exact stochastic simulation of chemical systems with many species and many channels. *J. Phys. Chem.*, 104:1876–1889, 2000.

[19] Daniel T. Gillespie. A general method for numerically simulating the stochastic time evolution of coupled chemical reacting systems. *J. Comput. Phys.*, 22:403–434, 1976.

[20] Daniel T. Gillespie. Approximate accelerated stochastic simulation of chemically reacting systems. *J. Chem. Phys.*, 115(4):1716–1733, 2001.

[21] Eric L. Haseltine and James B. Rawlings. Approximate simulation of coupled fast and slow reactions for stochastic chemical kinetics. *J. Chem. Phys.*, 117(15):6959–6969, 2002.

[22] Johan Hattne, David Fange, and Johan Elf. Stochastic reaction–diffusion simulation with MesoRD. *Bioinformatics*, 21(12):2923–2924, 2005.

[23] Markus Hegland, Conrad Burden, Lucia Santoso, Shev MacNamara, and Hilary Booth. A solver for the stochastic master equation applied to gene regulatory networks. *J. Comput. Appl. Math.*, 205(2):708–724, 2007.

[24] Andreas Hellander and Per Lötstedt. Hybrid method for the chemical master equation. *J. Comput. Phys.*, 227(1):127–151, 2008.

[25] Shev F. MacNamara. *Krylov and Finite State Projection Methods for Simulating Stochastic Biochemical Kinetics via the Chemical Master Equation*. PhD thesis, The University of Queensland, Australia, 2008.

[26] The Mathworks Inc. *Partial Differential Equation Toolbox*, 2.3 edition, 2017.

[27] Harley H. McAdams and Adam Arkin. It's a noisy business! Genetic regulation at the nanomolar scale. *Trends in Genetics*, 15(2):65–69, 1999.

[28] Johan Paulsson, Otto G. Berg, and Mans Ehrenberg. Stochastic focusing: Fluctuation-enhanced sensitivity of intracellular regulation. *Proc. Nat. Acad. Sci. USA*, 97(13):7148–7153, 2000.

[29] Christopher V. Rao and Adam P. Arkin. Stochastic chemical kinetics and the quasi-steady-state assumption: Application to the Gillespie algorithm. *J. Chem. Phys.*, 118(11):4999–5010, 2003.

[30] Y. Saygun. Computational stochastic morphogenesis. Master's thesis, Uppsala University, 2015. UPTEC F15 041.

[31] Mukund Thattai and Alexander van Oudenaarden. Intrinsic noise in gene regulatory networks. *Proc. Nat. Acad. Sci. USA*, 98:8614–8619, 2001.

A Stochastic chemical kinetics

In this section we briefly describe how reaction and diffusion events are modeled and how we obtain the diffusion rate constants when the domain is discretized using an unstructured mesh. For a more detailed introduction to the subject along with many additional references, consult, e.g., [11].

The computational core of URDME is based on the next subvolume method (NSM) [8]. Details concerning the actual simulation algorithms can be found in Appendix B.

A.1 Mesoscopic chemical kinetics

In a well-stirred chemical environment reactions are understood as transitions between the states of the integer-valued state space counting the number of molecules of each of D different species. The intensity of a transition is described by a *reaction propensity* defining the transition probability per unit of time for moving from the state x to $x + N_r$;

$$x \xrightarrow{\omega_r(x)} x + N_r, \tag{A.1}$$

where $N_r \in \mathbf{Z}^D$ is the transition step and is the rth column in the *stoichiometric matrix* N. Eq. (A.1) defines a continuous-time Markov chain over the positive D-dimensional integer lattice.

When the reactions take place in a container of volume Ω, it is sometimes useful to know that the propensities often satisfy the simple scaling law

$$\omega_r(x) = \Omega u_r(x/\Omega) \tag{A.2}$$

for some function u_r which does not involve Ω. Intensities of this form are called *density dependent* and arise naturally in a variety of situations [15, Ch. 11].

A.2 Mesoscopic diffusion

In the mesoscale model, a diffusion event is modeled as a first order reaction taking species S_l in subvolume ζ_i from its present subvolume to an adjacent subvolume ζ_j,

$$S_{li} \xrightarrow{a_{ij}\mathbf{x}_{li}} S_{lj}, \tag{A.3}$$

where $\mathbf{x}_{li}$ is the number of molecules of species l in subvolume i. On a uniform Cartesian mesh such as those used in MesoRD [22], the rate constant takes the value $a_{ij} = \gamma/h^2$ where h is the side length of the subvolumes and γ is the diffusion constant. In URDME we use an unstructured mesh made up of tetrahedra and the rate constants are taken such that the expected value of the number of molecules divided by the volume (the concentration) converges to the solution obtained from a consistent FEM discretization of the diffusion equation

$$u_t = \gamma \Delta u. \tag{A.4}$$

Using piecewise linear Lagrange elements and mass lumping, we obtain the discrete problem

$$u_t = M^{-1} K u \tag{A.5}$$

where M is the lumped mass matrix and K is the stiffness matrix. The rate constants on the unstructured mesh are then given by

$$a_{ij} = \frac{1}{\Omega_i} k_{ij}, \tag{A.6}$$

where Ω_i is the diagonal entry of M and can be interpreted as the volume of the dual element associated with mesh node i (see Figure A.1). For more details, consult [14].

The assumption made in the mesoscopic model is that molecules are well-stirred within a dual cell. These dual cells correspond to the cubes of the staggered grid in a Cartesian mesh.

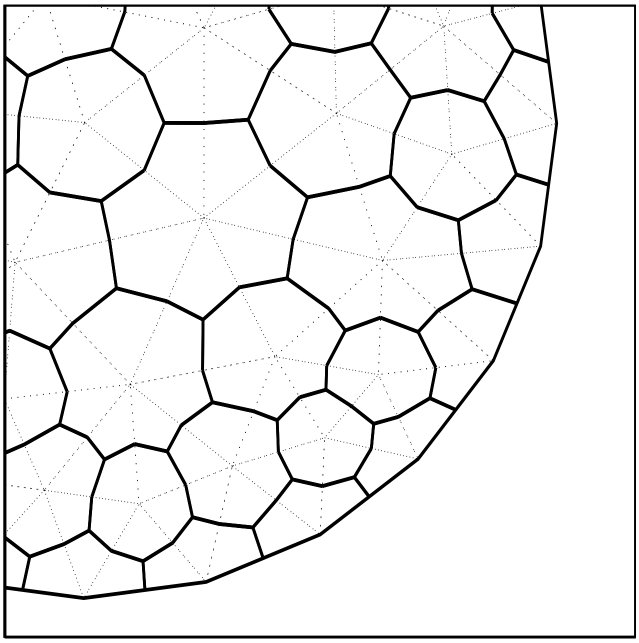

Figure A.1: A 2D example of an unstructured triangular mesh. The primal mesh is shown in dashed and the dual in solid. Within each dual element the system is assumed to be well-stirred, and molecules can jump from each dual cell to the neighboring ones.

B Algorithms

One of the most popular algorithms to generate realizations of the CTMC in the well-stirred case is Gillespie's direct method (DM) [19]. Several algorithmic improvements of this method exist, one of them being the next reaction method (NRM) due to Gibson and Bruck [18].

The underlying algorithm in URDME is the next subvolume method (NSM) [8]. The NSM can be understood as a combination of NRM and DM in order to tailor the algorithm to reaction-diffusion processes.

For reference, we first state below both DM and NRM and then outline NSM.

Algorithm 1 Gillespie's direct method (DM)

Initialize: Set the initial state $\mathbf{x}$ and compute all propensities $\omega_r(\mathbf{x}), r = 1, \ldots, M_{\text{reactions}}$. Also set $t = 0$.
while $t < T$ **do**
  Compute the sum λ of all the propensities.
  Sample the next reaction time (by inversion), $\tau = -\log(\text{rand})/\lambda$. Here and in what follows, 'rand' conveniently denotes a uniformly distributed random number in $(0, 1)$ which is different for each occurrence.
  Sample the next reaction event (by inversion); find n such that $\sum_{j=1}^{n-1} \omega_j(\mathbf{x}) < \lambda \, \text{rand} \le \sum_{j=1}^{n} \omega_j(\mathbf{x})$
  Update the state vector, $\mathbf{x} = \mathbf{x} + N_n$ and set $t = t + \tau$.
end while

Algorithm 2 Gibson and Bruck's next reaction method (NRM)

Initialize: Set $t = 0$ and assign the initial number of molecules. Generate the dependency graph G. Compute the propensities $\omega_r(\mathbf{x})$ and generate the corresponding *absolute* waiting times τ_r for all reactions r. Store those values in a heap H.
while $t < T$ **do**
  Remove the smallest time $\tau_n = H_0$ from the top of H, execute the nth reaction $\mathbf{x} := \mathbf{x} + N_n$ and set $t := \tau_n$.
  for all edges $n \to j$ in G **do**
    if $j \neq n$ **then**
      Recompute the propensity ω_j and update the corresponding waiting time according to

$$\tau_j^{\text{new}} = t + \left(\tau_j^{\text{old}} - t\right) \frac{\omega_j^{\text{old}}}{\omega_j^{\text{new}}}.$$

    else $\{j = n\}$
      Recompute the propensity ω_n and generate a new absolute time τ_n^{new}. Adjust the contents of H by replacing the old value of τ_n with the new one.
    end if
  end for
end while

Algorithm 3 The next subvolume method (NSM)

Initialize: Compute the sum σ_i^r of all reaction rates ω_{ri} and the sum σ_i^d of all diffusion rates $a_{ij}\mathbf{x}_{si}$ in all subvolumes $i = 1, \ldots, N_{\text{cells}}$. Compute the time until the next event in each subvolume, $\tau_i = -\log(\text{rand})/(\sigma_i^r + \sigma_i^d)$, and store all times in a heap H.
while $t < T$ **do**
  Select the next subvolume ζ_n where an event takes place by extracting the minimum τ_n from the top of H.
  Set $t = \tau_n$.
  Determine if the event in ζ_n is a reaction or a diffusion event. Let it be a reaction if $(\sigma_n^r + \sigma_n^d)\,\text{rand} < \sigma_n^r$, otherwise it is a diffusion event.
  if Reaction event **then**
    Determine the reaction channel that fires. This is done by inversion of the distribution for the next reaction given τ_n in the same manner as in Gillespie's direct method in Algorithm 1.
    Update the state matrix using the (sparse) stoichiometric matrix N.
    Update σ_n^r and σ_n^d using the dependency graph G to recalculate only affected reaction- and diffusion rates.
  else {Diffusion event}
    Determine which species S_{ln} diffuses and subsequently, determine to which neighboring subvolume $\zeta_{n'}$. This is again done by inversion using a linear search in the corresponding column of D.
    Update the state: $S_{nl} = S_{nl} - 1$, $S_{n'l} = S_{n'l} + 1$.
    Update the reaction- and diffusion rates of subvolumes ζ_n and $\zeta_{n'}$ using G.
  end if
  Compute a new waiting time τ_n by drawing a new random number and add it to the heap H.
end while
